# Development of sand-plastic composites as floor tiles using silica sand and recycled thermoplastics: a sustainable approach for cleaner production

**DOI:** 10.1038/s41598-022-19635-1

**Published:** 2022-11-07

**Authors:** Ashish Soni, Pankaj Kumar Das, Mohammad Yusuf, Hesam Kamyab, Shreeshivadasan Chelliapan

**Affiliations:** 1 Department of Mechanical Engineering, National Institute of Technology Agartala, Tripura, 799046 India; 2 Department of Petroleum Engineering, Universiti Teknologi PETRONAS, 32610 Bandar Seri Iskandar, Perak Malaysia; 3 Malaysia-Japan International Institute of Technology, Universiti Teknologi Malaysia, Jalan Sultan Yahya Petra, 54100 Kuala Lumpur, Malaysia; 4 Department of Biomaterials, Saveetha Dental College and Hospital, Saveetha Institute of Medical and Technical Sciences, Chennai, 600 077 India; 5 Engineering Department, Razak Faculty of Technology & Informatics, Universiti Teknologi Malaysia, Jalan Sultan Yahya Petra, 54100 Kuala Lumpur, Malaysia

**Keywords:** Engineering, Chemical engineering

## Abstract

Strict environmental concerns, depleting natural recourses, and rising demand for building construction materials have promoted scientific research toward alternative building materials. This research supports the idea of sustainability and a circular economy via the utilization of waste to produce value-added products. The research explored the potential of waste plastics and silica sand for developing thermoplastic composite as floor tiles. The samples were characterized by water absorption, compressive strength, flexural strength, and sliding wear. The morphological analysis of the sand-plastic interfaces was covered under the umbrella of this study. The maximum compressive and flexural strength were found to be 46.20 N/mm^2^ and 6.24 N/mm^2^, respectively, with the minimum water absorption and sliding wear rate of 0.039% and 0.143 × 10^–8^ kg/m, respectively. The study suggests the workability of the developed floor tiles in non-traffic areas of public places. Thus, the study provides a green building material through recycling waste plastics for sustainable development.

## Introduction

Plastics are widely used in households, industries, construction, automobiles, etc., replacing many conventional materials and products. Despite several remedial efforts, the use of plastic can’t be arrested due to its wide applications and beneficial properties. Global plastic production has increased significantly, and a considerable part is used for packaging. Plastic waste comprises 8–12% of the municipal waste stream, and approximately 190 million tonnes are generated annually^1^. In Australia alone, 2.24 million tons of plastic waste were generated in 2008, which comprised 16% of the municipal waste stream^2^. Together, informal and household sectors covered a generation rate of 6.5–8.5 million tons/year of plastic waste in India. Thus, plastic waste is considered one of the significant environmental problems due to its hazardous effects and difficulty of disposal. Factors such as population growth, low production costs, wide varieties, and applications result in an increased production of plastics^3^. As a result, a large quantity of plastic is discarded, and only a fractional part of it is recycled; thus, the effective management of plastic waste remains a global challenge for both developed and developing countries^4^. The traditional approach of managing plastic waste as a landfill is unsustainable and has given the scarcity of land in urban precincts. The disposal of plastic waste into landfills leads to remain locking up of valuable resources.

Consequently, new materials and energy are required for plastic production and introducing more hazardous wastes into the environment. An efficient waste management approach is required to manage such a large diversity of plastic in an efficient and environmentally friendly manner. Construction industries are increasingly seeking opportunities to recycle plastic waste as an alternative resource. Still, no such work is being conducted which demonstrates the development of sustainable polymer-based composites by using the distinct plastics waste and investigates the effects of compositions on the workability of the developed product. The polymer-based composite is applied to building construction materials such as floor tiles and pavements. The research demonstrates the development of eco-friendly thermoplastic composite materials like floor tiles. The study is imperative from a socio-eco-environmental point of view. The successful recycling of waste plastic for developing new materials would significantly improve the environmental conditions and reduce the demand for virgin quarry materials. Recycling plastic waste for developing more recent products is essential from a socio-eco-environmental point of view. It saves and sustains natural resources which can’t be replenished, decreases environmental pollution, saves energy and reduces the requirement for fresh raw materials for building materials. The study provides a direction for future research on sustainable polymeric composite materials.

### Solid waste management in the Indian scenario

**Figure 1:**
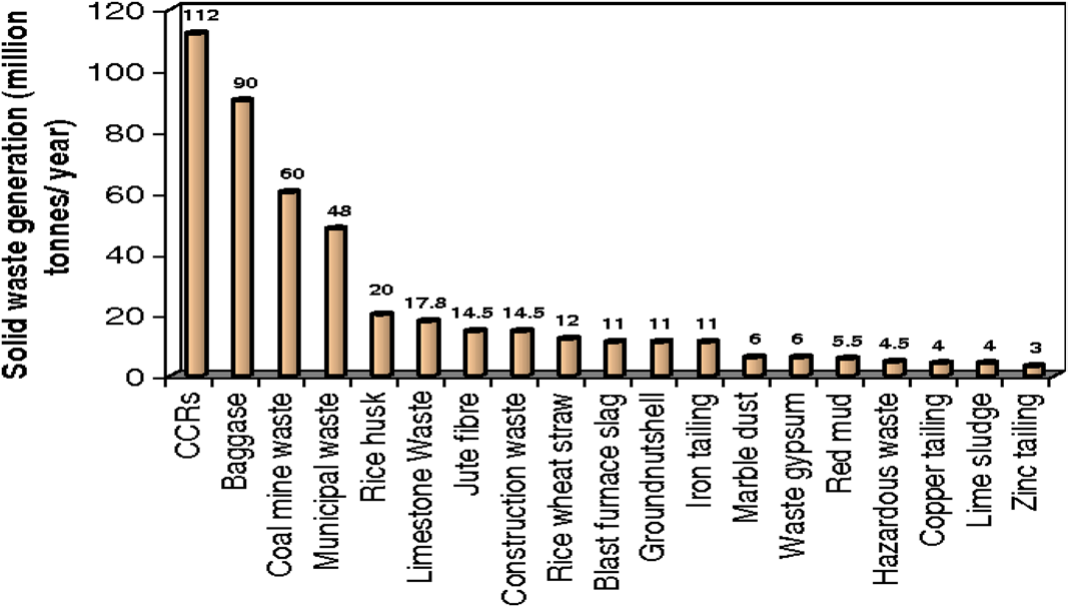
Solid waste generation in India (Million tones/year).

**Figure 2:**
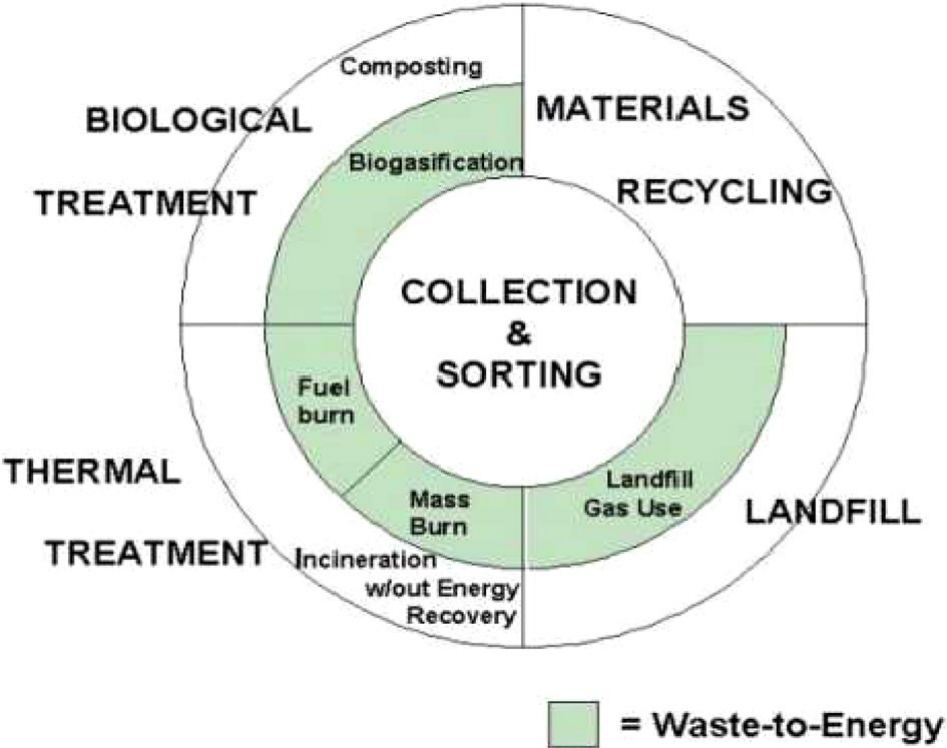
Elements of integrated solid waste management.

**Figure 3:**
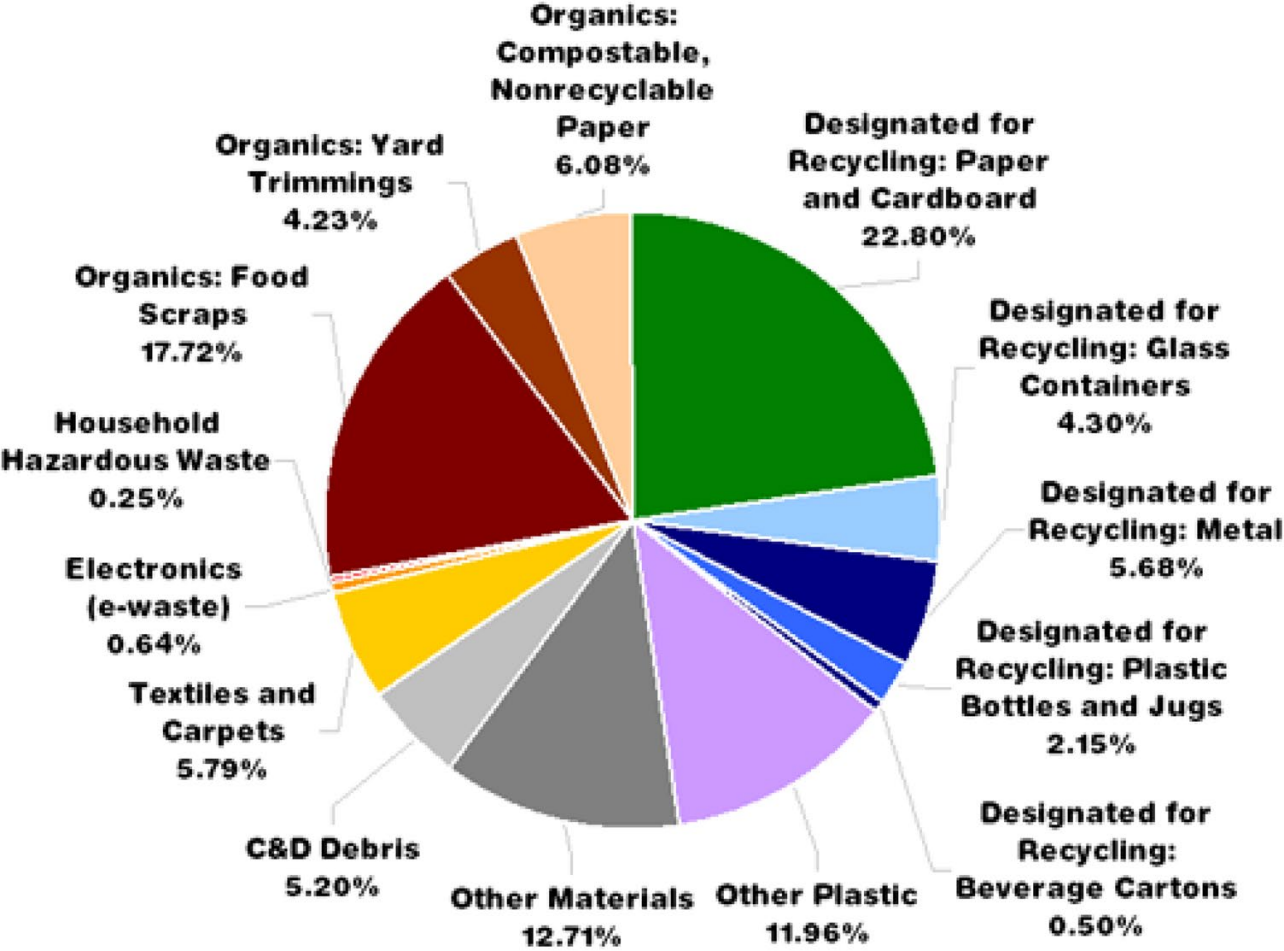
Recycling and utilization potential of solid waste.

This section discusses the sources of origination of solid waste and the worriment associated with its management^5^. The country’s vast population is liable for the mammoth generation of solid waste. Solid waste mainly comprises compostable items (51–53%), followed by recyclable (17–18%) with a moisture content of 46.76%. Figure 1 shows the rate of generation of different solid wastes in India^6^. The generated solid waste contains a C/N ratio of nearly 32 with a calorific value of about 1700–1800 kcal/kg. Still, only a fraction of the generated solid waste is used for composting, as shown in Fig. 2. Although plenty of technologies are available for the recovery of energy from waste and treatment of municipal solid waste, a significant part of the generated solid waste is unswervingly dumped over or into land in an undesirable manner. Therefore, municipal solid waste management has been found inadequate, ineffective, and challenging^7,8^. The report “Waste to Energy and Waste Management Market in India-2018” deeply examines the underlying opportunities associated with economies of scale, market trends, challenges and outlook of the waste and energy management industry^9^. The report suggests approaches to monitor the waste plastics and highlights the methods for diverting the waste plastics from landfills for safe disposal. Figure 3 shows the recycling potential for various solid wastes. The study forecasts the danger associated with solid waste and suggests measures for effective management of generating solid waste^10^. Some methods that play a pivotal role in managing plastic waste are synthetic lightweight aggregate technology, polymer concrete, etc., discussed^11–15^. Further, the studies suggest that the changes at the operation site affect industries engaged with the disposal of plastic waste and analyze some of the parameters to be executed in plastic manufacturing^16–18^. It was established that the collective effort of the public, government, and manufacturing industries is required to minimize the hazardous effect of waste plastics that would lead to green manufacturing in the building sector^19^.

### Challenges and issues for solid waste management

The significant challenges for solid waste management are associated with the different aspects of the waste sectors, including waste generation, inadequate waste collection, transport, treatment, and disposal processes. In the past few decades, there has been a rapid increase in the waste generation rate besides the poor waste collection practice. The logistic service for transportation of the generated waste is inadequate and inefficient. It requires overseeing the movement of the solid waste from the source to the sink. Inefficient waste logistic channels cause difficulties in managing the ingress and egress of solid waste from a waste dump to a treatment facility and vice versa. The waste-to-energy methods for energy recovery suffer from poor operational efficiency as about 2–3% of the energy is consumed to treat waste. An effective remedy to the problem is to change the mode of waste treatment from mechanical to biological. The availability of trained and certified personnel to manage the waste treatment protocols is mandatory. The destination of any waste is a junkyard, which also involves a cost-intensive process (e.g., handling, logistics, and environmental safety considerations).

### Impact assessment of solid waste

If agro-industrial wastes are utilized in various structural materials as an alternative to asbestos fibre, timber, and mica, a critical proportion of imperativeness can be saved^20,21^. The essential records to check the central focuses in Sustainable Solid Waste Management (SSWM) system are sullying decrease, saving imperativeness, and favourable social circumstances. Eventually, about 28% of the total energy resources are accounted for by building materials. Incorporating 25% of fly ash or 40% of blast furnace slag in Portland-pozzolana cement can save 30% of energy and result in a product of equivalent quality to that of original Portland cement.

Similarly, adding 25% fly ash with clay soil saves 15% energy for burnt clay bricks. Cement/resin bonded board and particle board can be used as a substitute for timber. The study reveals that around 20% of energy could be saved by introducing rice husk or groundnut for manufacturing particleboard. Plastic waste has a worthy potential for making various things concerning imperativeness age. Stringent authoritative standards increase the cost of waste exchange in multiple countries. Exact information on the entireties and sorts of waste conveyed by their motivation and objective isn’t extensively available. Given the characteristics of wastes and common dangers, multidisciplinary research work is critical in a planned manner to review and comprehend the potential employments of hazardous and non-hazardous wastes to comprehend their motivating force for various applications in a natural and welcoming way.

### Statement of problem

Industrialization, rapidly growing population, urbanization, increased consumption levels, etc., are the consequences of an exponential solid waste generation rate increase. Table 1, given below, summarizes the generation and handling of solid waste in Mega tons per day (MT/D) in urban areas of Indian states. The global waste management report showed an increase in the worldwide generation of municipal solid waste was raised by 37.3%, equivalent to the rise of 8% per year from 2007 to 2011. By 2015, the generation rate of solid waste reached 1.3 billion tons/year, which increased by 13% annually. It further projects that around 19 billion tonnes of solid waste are expected to be generated annually by 2025.

**Table 1:** Generation and handling of solid waste in urban areas of different Indian States^25^.

State	Waste generation (MT/D)	Waste collected (MT/D)	Waste treated (MT/D)	Landfilled (MT/D)	Treatment methods
Andhra Pradesh	6525	6331	500	143	Vermi-composting, biomethanization, Wt E (63 MW generation capacity)
Arunchal Pradesh	181	11	500	143	Vermi-composting, biomethanization, WtE (63 MW generation capacity)
Assam	1134	6336	200	143	WtE
Bihar	1192	6336	200	143	WtE, landfilling
Chandigarh	340	360	200	143	WtE, landfilling
Chhattisgarh	1959	2036.97	828.18	1294.97	Composting, vermi-composting,
Delhi	10,500	2036.97	828.18	1294.97	Landfilling, WtE (52 MW generation capacity), composting
Goa	240	2036.97	828.18	1294.97	Landfilling
Gujrat	10,145	10,480	2565	7730	Vermi-composting, biogas, RDF, WtE
Haryana	4514	3102.51	188	2163.18	Composting, landfilling
Jammu & Kashmir	1792	1388.7	3.45	425	Composting, landfilling
Jharkhand	2451	3238.65	65	3179.7	Composting, landfilling
Karnataka	10,000	7716	3584	3946	Landfilling, composting, vermi-composting, biogas, RDF
Madhya Pradesh	6424	7716	3584	3946	Composting, vermi-composting, WtE
Maharashtra	22,570	21,867.27	6993.2	14,993.07	Composting, vermi-composting, WtE (16 MW generation capacity), biogas, bio-reactor landfill
Meghalaya	268	156	36	120	Composting, vermi-composting
Orissa	2460	2283.9	30	120	Composting, vermi-composting
Pondicherry	495	513	10	503	Composting, vermi-composting
Punjab	4100	4435	3.72	3214	Landfilling
Tamil Nadu	15,547	210	3.72	207	Biomethanation, WtE, RDF, compost, recycling
Tripura	421	368.2	250.4	164.4	Composting, vermi-composting
Telangana	7371	6625	3175	3050	RDF, WtE (30 MW generation capacity)
Uttar Pradesh	15,547	11,394	1857	3050	Composting, RDF
Uttarakhand	1400	917.89	1857	3050	Composting, RDF
Others	18,050	917.89	1857	3050	Composting, RDF
Total	1,45,626	67,903.8	20,289	41,133.3	

Furthermore, by 2047, the MSW generation in India is expected to reach 300 MT^21^. Among the generated solid waste, 8–10% have low biodegradability and can take more than 500 years to decompose, thus requiring vast land for disposal, making it a remarkable factor for socio-economic development and environmental health^22^. In another context, shelter being a necessity for human beings is thus, creating a massive demand for fresh raw materials in the sector of construction. Hence, recycling waste plastic for building materials development is considered a viable solution^23,24^.

## Statement of novelty

The development of an effective recycling method is considered one of the most urgent requirements to overcome the burden of plastic waste around the globe. The waste plastics are being used as a matrix for developing polymer-based composites found in building construction materials to improve the recycling practice. However, the workability of such a composite is unexplored. Moreover, there is a shortage of available data for the characteristics of plastic-sand composites and their responses to the given properties. Despite their great potential, this research gap has hampered the application of polymeric composites to a wide range of building construction materials. The comprehensive study of plastic waste highlights the opportunities of plastic waste for the socio-eco-environmental development of a nation. Moreover, the experimental results revealed the suitability of the polymer-based composite materials for application as floor tiles. The research would assist in the development of value-added products through the recycling of waste plastics.

## Related work

**Figure 4:**
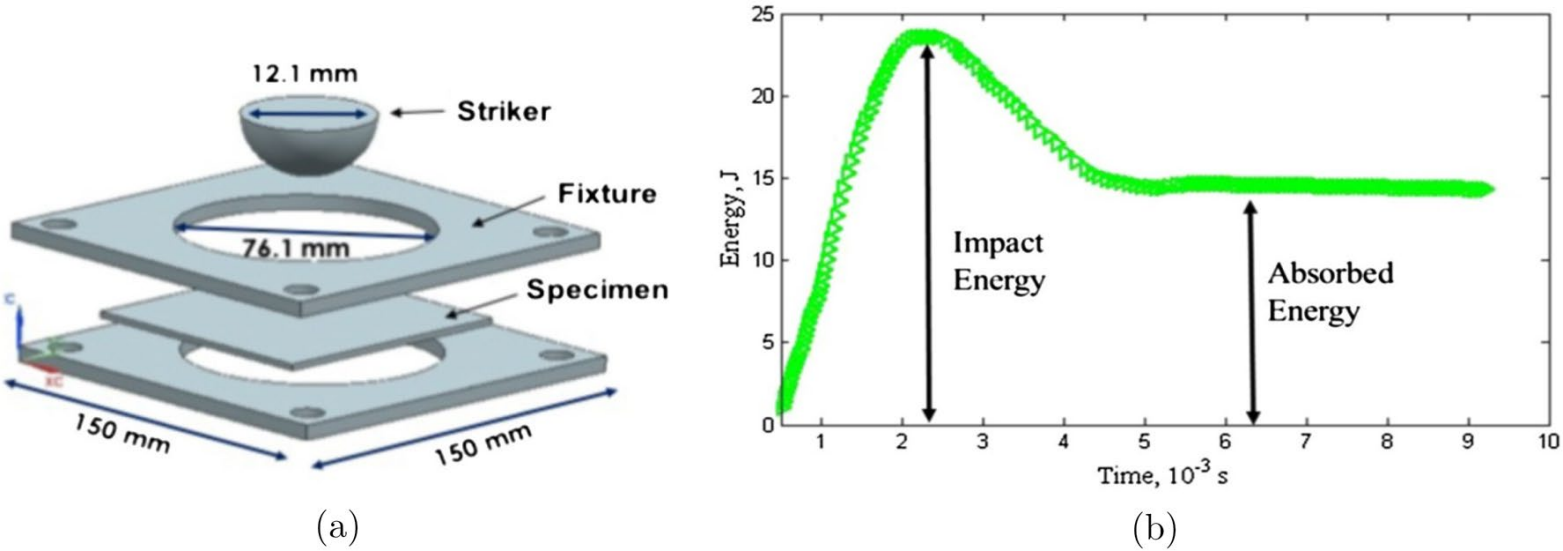
(**a**) Schematic of the impact fixture. (**b**) Typical energy-time response to dynamic impact loading.

**Figure 5:**
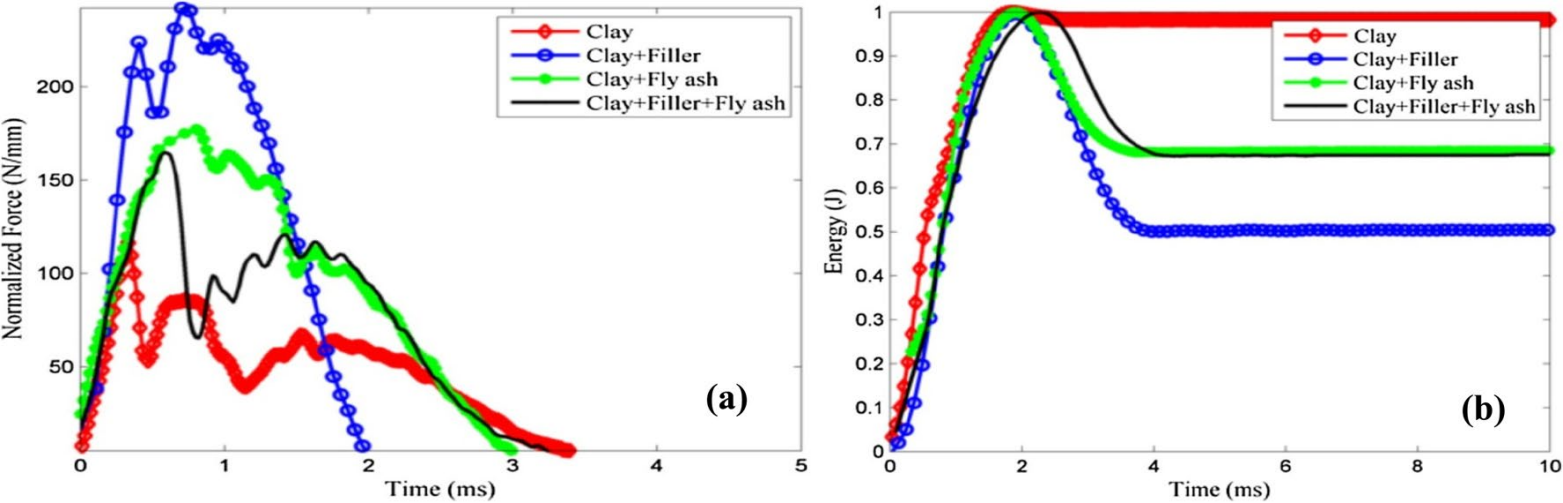
(**a**) Normalized force–time response for one set of samples at 1 J. (**b**) Energy-time response for one set of samples at 1 J.

Although various methods are available for recycling solid waste, the effective management of the generated solid waste is still restricted. The recyclable materials considered in the three significant areas are economy, compatibility and material properties, and recycling which is beneficial when the recycled product is cost-effective with desirable properties. Table 2 shows the various recyclable hazardous wastes with their generating sources^26^. The recyclable materials will be more combative in a condition where the problem of fresh raw materials and landfilling subsists. This section excavates (i) various technologies of waste recycling and their viability; (ii) identification of upcoming opportunities for solid waste management; (iii) consideration of ethical, economic, and technological points for recycling of plastic in India; (iv) the organizations working for preventing and controlling pollution due to solid waste and various schemes for waste management (v) reviews the solid waste utilized for the fabrication of roof tiles along with characterizations and experimental validation. The study aims to achieve an adequate strength of concrete by using waste ceramic as an alternative to coarse aggregates. Concrete was made with waste tiles of varying proportions. It was found that the ceramic waste should be deglazed, and a lower cement ratio is adopted to achieve the desired strength. The study for manufacturing roof tiles using industrial waste along with natural fibres demonstrated that by mixing fibrous composites, better mechanical properties and a weight reduction could be achieved^27,28^. The academicians had worked on the fibres from the PET bottle wastes to improve the flexural capacity of the concrete^29^. The damping properties of concrete tile with plastic fibres of 10% and 15% were found to be better than conventional concrete. Liang et al., experiments for the development of eco-friendly tiles by utilizing waste plastics and sand showed that adding sand enhances its strength, thus making it more durable to withstand the temperature and exploring its suitability for use in a terrace. Moreover, the compressive strength and water absorption were comparable with normal tiles^30^. Lopez et al., investigated ceramic tiles’ production by mixing blast furnace slag and clay and further characterized to obtain a suitable mixture and optimum temperature^31^. Li et al., estimated the durability of the wood-plastic composites by conducting the absorption test, weathering by accelerated xenon arc lighting and freeze–thaw cycling^32^. De Silva et al., describe the production of sheet moulded compound roof tiles. The values for tensile strength and elastic modulus were 30 ± 3 MPa, and 10 ± 1 MPa and cracks were found at 94 kg of static loading, which is quite sufficient to carry the load of a normal person^33^. Madhi et al. discussed roof tiles’ preparation using rice husk, M-sand, and soil. The test results suggest that fine rice husk particles in an appropriate proposition can successfully replace M-sand^34^. Amaral et al., describe the production of floor tiles using waste material produced during the cutting of ornamental rocks. The tiles were found to meet the ISO 13006 standard^35^. Geng et al., evaluate the possibility of making ceramic tiles by utilizing waste wood as a forming agent. The new technology contributes to sustainable construction and increases the range of application of stoneware tiles^36^. Novais et al., compared the properties of roof tiles prepared using waste plastic and rubber with ceramic roof tiles^37^. Makarichi et al., work for ceramic roof tiles by using sewage sludge in ceramic mass and suggest the addition of 4% of dry sludge to meet the standards^38^. The flexure strength was found to behave inversely with sludge content^39^. Castellanos al. demonstrated the fabrication of roof tiles by utilizing fly-ash cenospheres to improve damage against impact loads. To achieve this, the four types of composites, clay + filler, clay + fly-ash, and clay + filler + fly ash, were taken and evaluated under a dynamic impact test, as shown in Fig. 4a,b. Figure 5a,b shows the force–time response for one trial of soil, mud + filler, earth + fly-garbage, and mud + fly-flotsam and jetsam + filler composites each for depiction^20^. Zhou et al., described the preparation of hardened tiles by using waste Phosphogypsum as raw material through an intermittent pressing hydration approach to prepare tiles^40^. Ilankoon et al., review the challenges associated with the generation of e-waste and strategies for management^41^. Huang et al., reported the frost behaviour of manufactured roof tiles by performing freeze testing and calculating the durability index due to the change in density of the water^42^. Talni et al., elaborate on the waste management techniques for recycling plastic in concrete^43^. A study was carried out about the consequences of plastic waste in the environment^44,45^. Vinayagamoorthy et al., proposed using plastic waste for sustainable development where waste plastics were incrementally substituted, and physical properties were determined^46^. The researcher concentrates on using molten scrap plastics and broken glass to produce tile^47^. It is shown that composite tiles can be successfully produced by using waste plastic bags which provides a new approach to plastic waste management. Furthermore, experiments were conducted to develop mortar using PVC waste materials as a mortar composition. The improvement in flexural rigidity and tensile strength by using PVC plastic waste in the mortar was demonstrated^48^. Turku et al., experimented with using plastic bottles with concrete to ensure their suitability in the masonry unit. The literature paves the way for partial substitution of the waste plastic to be used with concrete^49^. The work involves the utilization of low-density polyethene with the incorporation of sawdust in proportions to evaluate the properties like the strength of compression, flammability, etc. Turku et al., demonstrate the use of plastic waste in developing composite floor tiles^50^.

**Table 2:** Sources and types of generated recyclable hazardous wastes^26^.

S. no.	Processing activities	Types of generated waste
1	Production or use of zinc, zinc oxide	Zinc ashes, dross, filter cake, jarosite
2	Production and use of copper oxide, Electro-refining and winning operations in copper smelter	Exhaust dust, residues, sledges
3	Iron and steel producing industries	Dust, slag, sludge
4	Production of aluminum	Filtered material, cathode residues
5	Production of asbestos and product containing asbestos	Residue containing asbestos
6	Metal surface treatment and hot dip galvanizing	Sludge and residue with acidic alkali
7	Production of paints, dyes, and pigment	Blasting material contaminated with coating residues, sludge from wastewater purification in production processes
8	Production of made with silicones	Residue containing silicone
9	The activities for treatment of solid waste like Incineration, distillation, separation, and concentration	Flue gases, ash and burned residue
10	Leather tanning and processing of waste leather	Sludge and residue of chromium and leather forming waste
11	Refinery producing solvents	Residue as solvent

## Methodical approach

The present research begins with a literature survey that summarizes the waste management practices from generation to utilization of various solid wastes for constructive application in an economical and environmentally friendly manner. The present work demonstrates the manufacturing of plastic-sand composite materials like floor tiles by using different kinds of waste plastics (including low-density polyethene, high-density polyethene, and polyethene terephthalate) as a binding material in replacement of cement with the incorporation of natural sand. The plastics available in various forms are found to have binding quality with other remarkable properties like lightweight, non-corrosive, toughness, durability, etc., thus making them suitable for use as a raw material for construction^51^. The test results on physical, mechanical, and tribological properties suggest its application as floor tiles in non-traffic areas of public places is potent for mitigating the problem related to the management of solid waste, thus improving the environmental condition. The study examines the need for future scopes of investigations for establishing better-mixing proportions, compositions, and process variables.

## Development of samples

### Materials

In the present work, waste plastics of distinct types, including low-density polyethene, high-density polyethene, and polyethene terephthalate, are used as binding agents in the replacement of cement for the development of floor tile samples. The commercially available silica sand having a grain size of 150 µm is taken as filler material. The values for bulk density and specific gravity of the silica sand are 1730 kg/m^3^ and 2.65, respectively, at ambient conditions. The properties of the waste plastics are listed in Table 3.

**Table 3:** Typical properties of the waste plastics.

S. no.	Property	Low-density polyethylene (LDPE)	High-density polyethylene (HDPE)	Polyethylene terephthalate (PET)
1	Density (g/cm^3^)	0.915–0.92	0.94–0.96	1.39
2	Tensile modulus of elasticity (MPa)	200–400	600–1400	3500
3	Yield stress (MPa)	8–10	18–30	90
4	Melting temperature (°C )	105–118	126–135	245
5	Water absorption at 23 °C (%)	< 0.05	< 0.05	0.25

### Method

**Figure 6:**
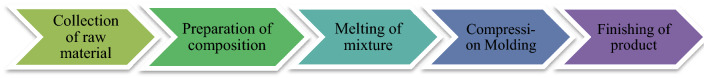
Process flow chart for development of specimens.

**Figure 7:**
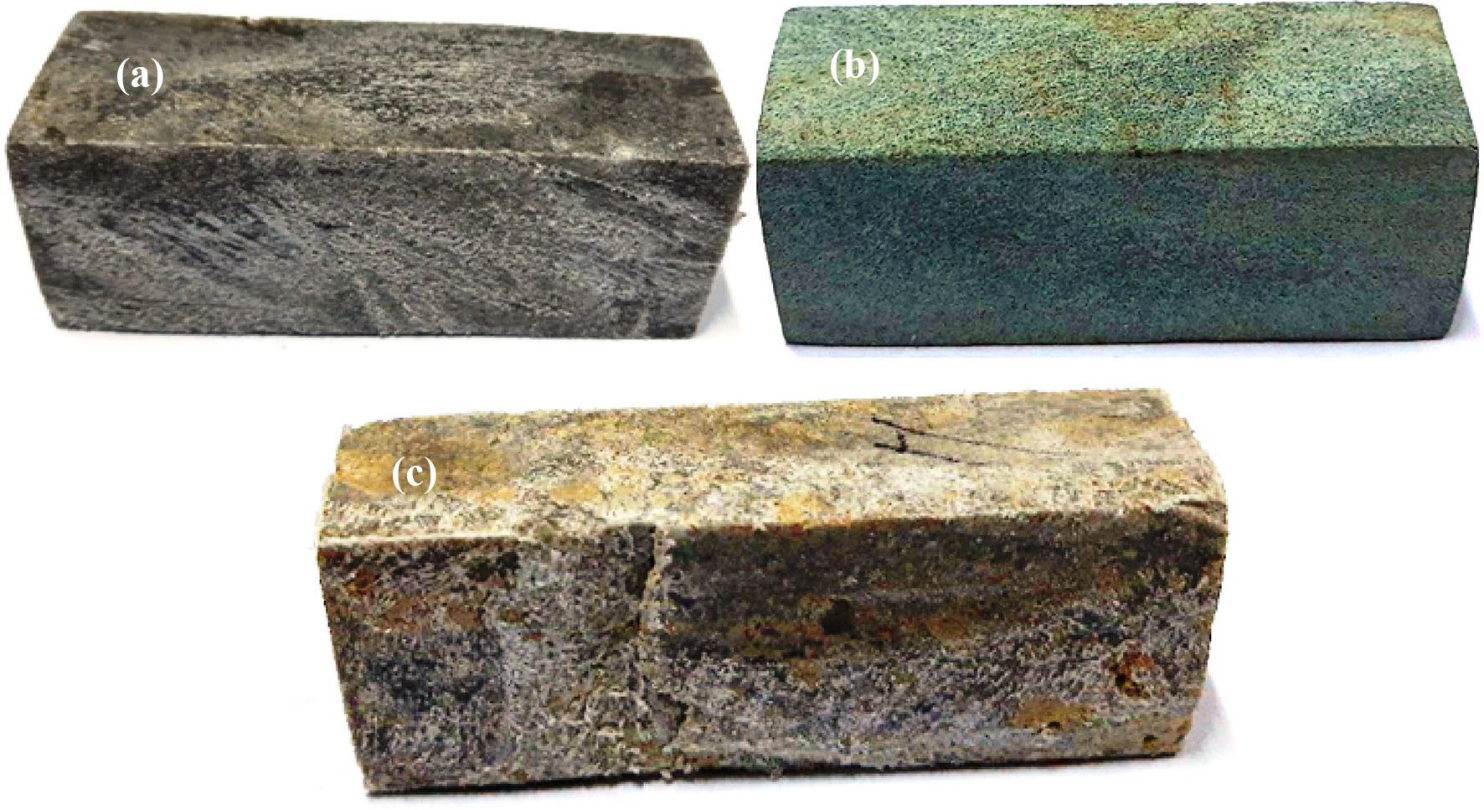
Images of prepared specimens (**a**) 50% of low-density polyethylene, (**b**) 50% of high-density polyethylene, (**c**) 50% of low-density polyethylene and 20% polyethylene terephthalate.

Figure 6 shows the process flow chart for the preparation of specimens. Plastic bags are initially collected from different waste collecting points in the municipality. The collected plastics were cut, washed to remove impurities, and dried thoroughly. The type of plastic is identified through the RGB method, which uses the three primary or basic colours, namely red (R), green (G) and blue (B). The main components of the method include a computer system, a webcam, and an integrated automatic system to identify the plastic based on its colour values. The processed waste plastics are then shredded into small 10–15 mm pieces. The shredded plastic waste is mixed with sand as per the composition given in Table 4. The mixture is heated at an elevated temperature up to the melting point of the plastics. It is mixed continuously until a homogenous blend of sand, and plastic waste is formed. The sample was cast through the static compaction technique under the constant pressure of 20.7 MPa, taking the mould size of 150 × 150 × 50 (mm). The range of shrinkage allowances (%) for the casting of samples S1, S2 and S3 were 2–4%, 1.5–4% and 0.2–3%, respectively. A similar procedure is followed for casting the remaining samples under the same pressure. Once the mould is obtained, its surface is finished, and the required dimensions taking tolerance of $\pm$ 1.0 mm are prepared for evaluation. Figure 7a–c gives the images of the floor tile samples prepared by following the composition as proposed.

**Table 4:** Compositions of composites.

S. no.	Specimen	Weight (%) of low-density polyethylene	Weight (%) of high-density polyethylene	Weight (%) polyethylene terephthalate	Weight (%) of sand particles
1	S1	50	–	–	50
2	S2	–	50	–	50
3	S3	50	–	20	30

### Structural analysis

**Figure 8:**
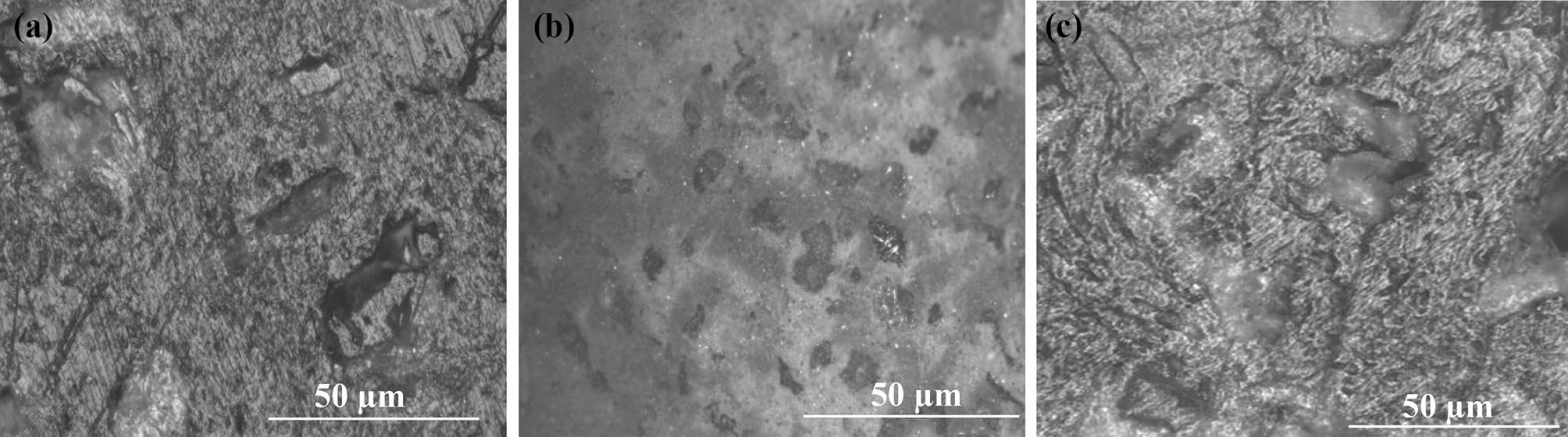
Surface image of specimens (**a**) 50% LDPE, (**b**) 50% HDPE, (**c**) 50% LDPE and 20% PET.

To study the homogeneity and uniformity of the compositions, the structural analysis was conducted using an optical microscope of Leica DMI 3000 M. The microscopic images of the specimen’s surfaces are shown in Fig. 8a–c. An intact morphology with completely encapsulated and mechanically bonded fillers into the plastic matrix was observed, thus providing high strength to the developed composites. Figure 8c shows a non-homogenous morphology of the surface; the difference in properties of low-density polyethylene and polyethylene terephthalate could be the possible cause. The specimen with high-density polyethylene and sand as composition is shown in Fig. 8b, demonstrating that the matrices fill the surface irregularities and hold the fillers together by forming a robust interfacial bond between the filler and matrix, thus providing sufficient strength.

Moreover, no holes are visible over the surfaces, which indicates the complete encapsulation of filler particles with the binding material. The obtained result for mechanical strength elaborates on its viscoelastic behavior. The behavior for mechanical strengths was analogous to studies made for elastomeric systems where the filler particles reinforced the matrix by diverting the rupture path, thus increasing the required energy for crack propagation^47^.

## Experimental evaluation of properties

The evaluation of properties is important for identifying the performance of the prepared specimens and establishing the workability of the developed composites. The developed floor tiles samples are evaluated for physical, mechanical, and tribological properties in water absorption, compressive strength, flexural strength, hardness, friction force, coefficient of friction, and sliding wear rate.

### Water absorption

The water absorption determines the moisture content of the floor tiles. It is expressed as a percentage of dry weight. The water absorption was evaluated by a 24 h immersion procedure as per the ASTM D 570-95 standard test method for water absorption^52^. The test for water absorption is performed by taking a sample size of 75 mm × 30 mm × 30 mm. The test identifies the suitability of floor tiles in different ambient conditions. It indicates the degree of internal porosity, water holding capacity, and quality of a material. The water absorption indicates the moisture content, equivalent to the difference between the wet and dry weight of the specimen divided by the weight of the dry specimen and multiplied by 100 to express it as a percentage. The Eq. (1) gives the formula to find the water absorption of the specimen.

1 $$Water \,absorption \left(\%\right)=\frac{Wet\, weight -Dry \,weight}{Dry \,weight } \times 100.$$

Here, the reduced-size prepared specimens are immersed in water for 24 h at 23 °C and weighted using a Mettler balance. This gives the wet weight of the specimen. The specimens are taken out and put into an oven for a specified time at an elevated temperature, then allowed to cool in a desiccator. Immediately after cooling, the specimens are taken out and reweighted, which is the dry weight of the specimen.

### Mechanical strength

**Figure 9:**
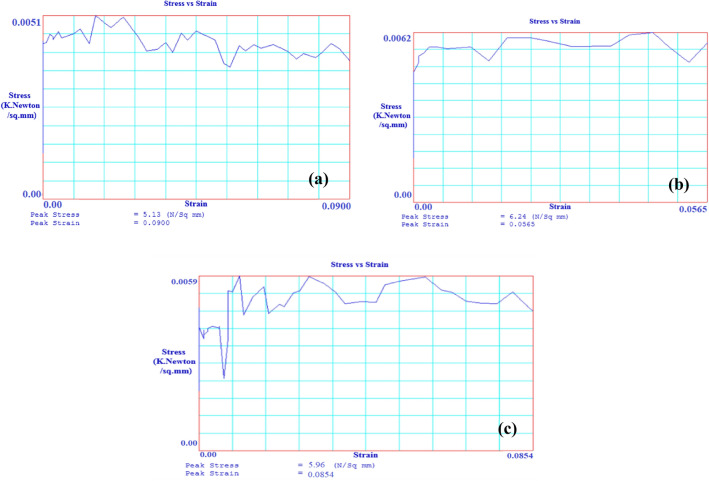
Image of stress v/s strain graph (**a**) S1, (**b**) S2, (**c**) S3.

The compressive strength indicates the stability of material against external forces. The prepared specimens’ compressive and flexural strength evaluations were carried out using a universal testing machine, Model No. HL 590 20 has a capacity of 600 KN. The specimens of size 50 mm × 50 mm × 50 mm are prepared per the test requirements. The compressive strength of the specimen was evaluated by placing the samples parallel to the surface between the compressive plates to provide the compressive load at a uniform rate. The three-point bending or flexural strength was evaluated using the same machine with different specimens and loading conditions. The specimens of standard size of a 75 mm × 10 mm × 10 mm for the flexural test are kept in the form of a cantilever beam, and a bending load is applied in the middle of the specimens to evaluate the value of flexural strength as per ASTM C1186-08 standard test for flexural strength^53^. The maximum load is recorded along with the stress–strain data given in Fig. 9a–c.

### Sliding wear

**Figure 10:**
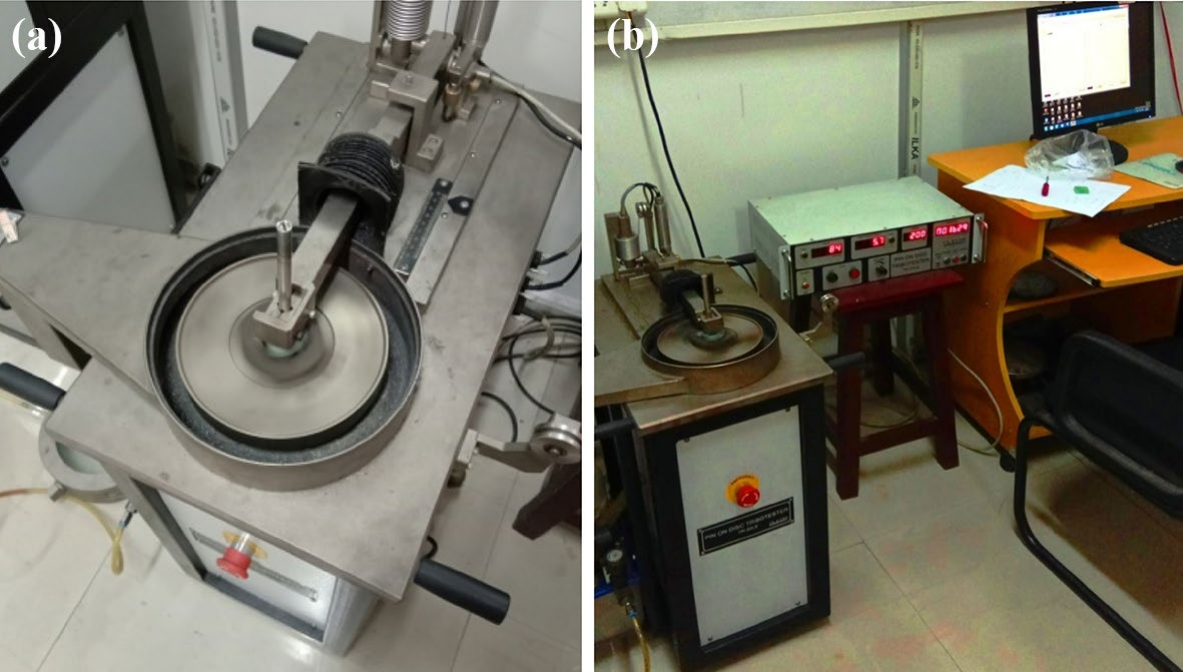
Wear test (**a**) sliding disc, (**b**) pin on disc setup.

Wear resistance is the surface property that resists wearing during rubbing and is the characteristic of an engineering material that depends on load condition, speed of relative motion, hardness of the material, ambient conditions, and the presence of foreign material. The wear rate (kg/m) is expressed as loss in weight (kg) divided by the sliding distance (m). In the present work, the evaluations for the sliding wear rate are carried out using a Pin on disc Tribo tester built by Ducom Instruments Pvt. Ltd., as shown in Fig. 10a,b. The wear test is performed by taking a sample of 30 mm × 30 mm × 10 mm, which is well surface finished to avoid any irregularity in the surface. The specimen ball is made of 100 Cr steel, has a diameter of 6 mm and is fixed to the holder above the rotating disc. The tests are performed under a load of 1, 3 and 5 kgf. The rotational speed is kept constant at 200 rpm for the test duration of 1800 s. The track diameter is 12 mm for the sliding distance of 226 m. The weight loss is calculated by subtracting the initial and final weight. The load on the arm pushes it to maintain contact with the disc during the sliding wear of the specimen. The movement of the arm is sensed by the sensors provided below the arm and generates signals, which give the value of maximum wear. A similar procedure is followed to evaluate the wear rate for the given specimens.

## Results and discussion

**Figure 11:**
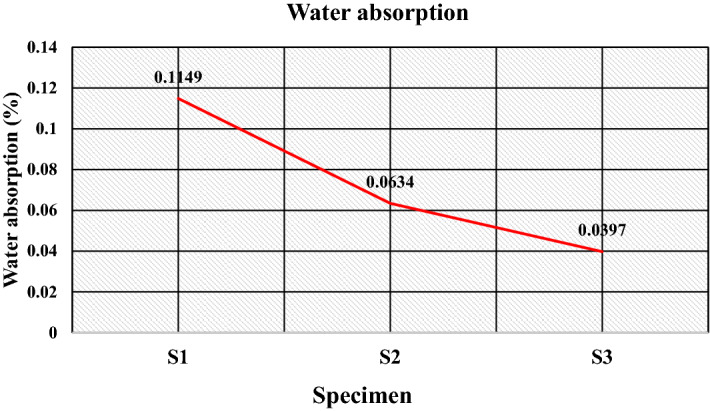
Water absorption (%) of the specimens.

The resulted values of water absorption for the developed samples are listed in Table 5. The results for water absorption show that the water absorption for the prepared specimens is less than 0.5%, as Fig. 11 illustrates, which suggests the suitability of the developed composites for use as floor tiles. The water absorption depends on the porosity present in the interior as well as in the surface layer of the aggregate^54^. Moreover, the ratio of open to closed porosity and interconnectivity between the pores at the surface and inner pores of the structure plays a significant role in water absorption^37^. The reduction in water absorption for the composite having 50 wt% of high-density polyethylene and 50 wt% of sand as the specimen S2 compared to the composite with 50 wt% low-density polyethylene and 50 wt% of sand as specimen S1 can be attributed to relatively lower porosity which further reduces the composite having 50 wt% low-density polyethylene, 20 wt% of PET and 30 wt% of sand as specimen S3. Moreover, when substituting a part of the natural sand with PET (with a different granulometry), the latter creates a proper and different porosity to the sand created by the sand due to its planar and elongated structure. This gives the minimum absorption of 0.0397%.

**Table 5:** Properties of the developed composites.

S. no.	Specimen	Water absorption (%)	Compressive strength (N/mm^2^)	Flexural strength (N/mm^2^)	Hardness (HV)
1	S1	0.1149	44.50	5.13	32
2	S2	0.0634	46.20	6.24	44
3	S3	0.0397	20.81	5.96	37

**Figure 12:**
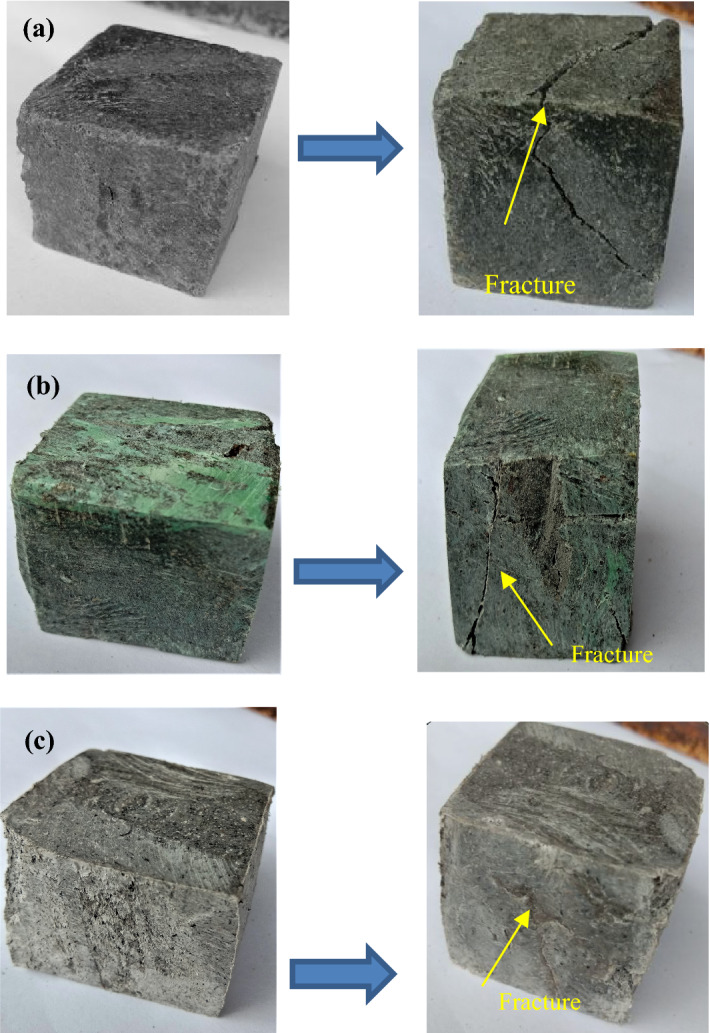
Image of specimen before and after compression test (**a**) S1, (**b**) S2, (**c**) S3.

**Figure 13:**
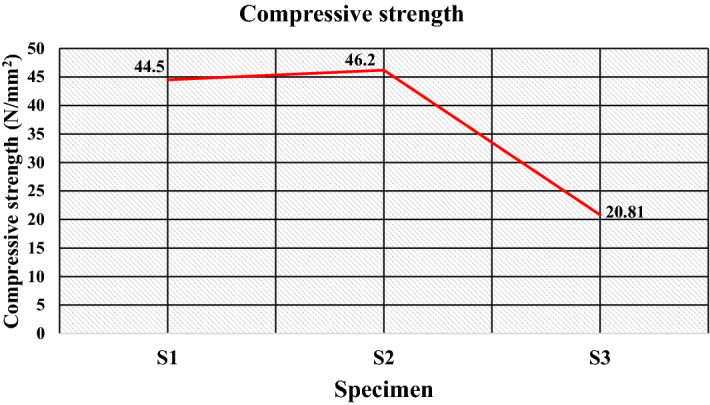
Compressive strength of the specimens.

The compressive strength indicates the resistance against the external compressive load. Figure 12a–c gives the images of the test specimens before and after the compression test. It was established that impact and abrasion resistance is associated with compressive strength^47^. A dissimilar tendency for compressive strength with composition is obtained, as shown in Fig. 13. The filler/matrix ratio influences the strength of the developed composite floor tile samples. The maximum compressive strength value is found for the composite having a composition of 50% high-density polyethylene and 50% silica sand for specimen S2 of 46.20 N/mm^2^ (as given in Table 5). The sufficient surface area provided by fillers and the higher strength of high-density polyethylene compared to low-density polyethylene favors an optimum compressive strength^55^.

Further, the compressive strength reduces to 44.50 N/mm^2^ for the composite with 50 wt% of low-density polyethylene and 50 wt% of sand particles as the specimen S1. The properties of the matrix as low-density polyethylene are responsible for the reduction in compressive strength, which further reduces to 20.81 N/mm^2^ for the specimen S3 having compositions of 70 wt% of waste plastics (50 wt% as low-density polyethylene and 20 wt% as PET) and 30 wt% of silica sand due to the insufficient surface with reduced fractions of filler particles.

**Figure 14:**
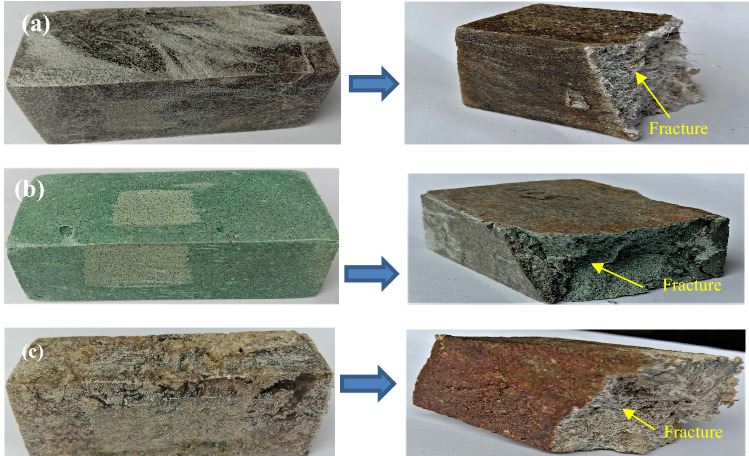
Image of specimen before and after flexural test (**a**) S1, (**b**) S2, (**c**) S3.

**Figure 15:**
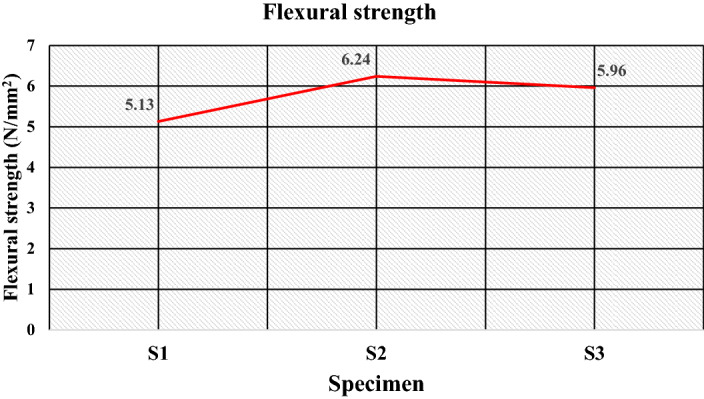
Flexural strength of the specimens.

Figure 14a–c gives the images of the fracture specimens due to flexural. The flexural strength values for the developed samples are given in Table 5, which shows the flexural strength values range from 5.13 to 6.24 N/mm^2^. The maximum strength was found to be 6.24 N/mm^2^ for the specimen S2 having 50 wt% of high-density polyethylene and 50 wt% of sand particles. The plots for the flexural strength in Fig. 15 show the decrease in the flexural strength value to 5.13 N/mm^2^ with the replacement of high-density polyethylene by low-density polyethylene, i.e., for the specimen S1 due to the lower strength of low-density polyethylene.

**Figure 16:**
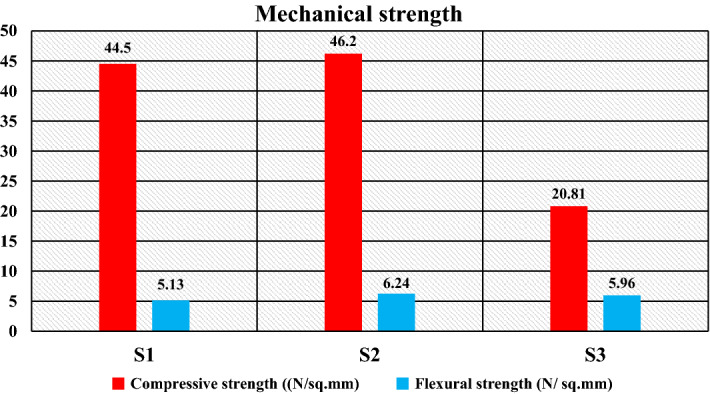
Mechanical strength of the specimens.

Further, specimen S3 has a composition of 70 wt% plastic wastes (50 wt% low-density polyethylene and 20 wt% PET) and 30 wt. % silica sand particles have insufficient surface area due to the reduced fraction of filler. Still, a significant portion of strength is compensated due to PET’s enhanced properties (as shown in Table 3). This gives the value of flexural strength of 5.96 N/mm^2^. The study of the flexural strength suggests that the incorporation of PET improves flexural strength and shows better flexural strength can be achieved by incorporating sand particles as fillers. For a comparative study of mechanical strength, the compressive and flexural strength results are plotted as shown in Fig. 16, establishing that the developed specimens have adequate mechanical, i.e., compressive strength and flexural strength, making them suitable for use as floor tiles. Moreover, the mechanical strength was observed due to the addition of PET in the replacement of silica sand, which also reduces the requirement for filler material.

**Figure 17:**
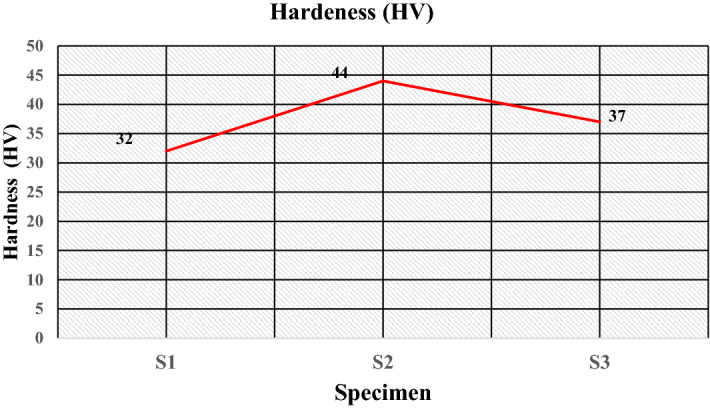
Hardness of the specimens.

The Hardness (HV) of the developed samples is evaluated using a Vickers hardness tester. The resulting hardness values are given in Table 5, which shows the hardness of the samples S1, S2 and S3 as 32, 44, and 39 (HV), respectively. The plot for the hardness is given in Fig. 17; It indicates that the value of hardness increases from 32 to 44 (HV) with replacements of 50 wt% of LDPE by HDPE, and then due to the reduced fraction of hard sand particles in the sample S3, the value of hardness decreases to 37 (HV).

Investigations of the tribological properties such as friction force, coefficient of friction, and wear rate are carried out. The resulted values of the tribological properties of the composite samples are given in Table 6.

**Table 6:** Tribological properties of the developed composites.

S. no.	Specimen	Friction force	Coefficient of friction (µ = F/R)	Wear at sliding distance of 226 m (kg)	Wear rate at 1 kgf (kg/m)	Wear rate at 3 kgf (kg/m)	Wear rate at 5 kgf (kg/m)
1	S1	5.4 N	0.11	32 × 10^–8^	0.885 × 10^–8^	0.143 × 10^–8^	13.926 × 10^–8^
2	S2	3.9 N	0.0795	1833 × 10^–8^	2.212 × 10^–8^	8.133 × 10^–8^	7.926 × 10^–8^
3	S3	9.2 N	0.187	292 × 10^–8^	2.655 × 10^–8^	1.291 × 10^–8^	3.978 × 10^–8^

**Figure 18:**
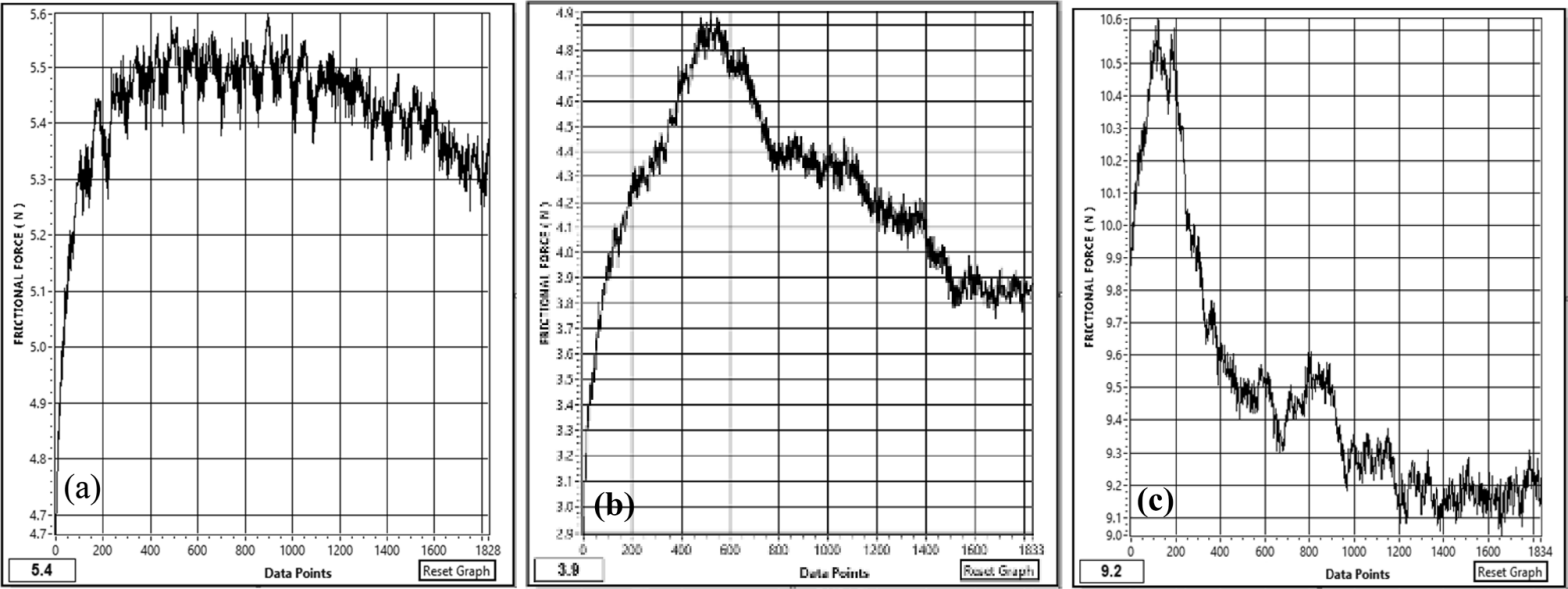
Friction Force of the specimens at data point (**a**) S1, (**b**) S2, (**c**) S3.

**Figure 19:**
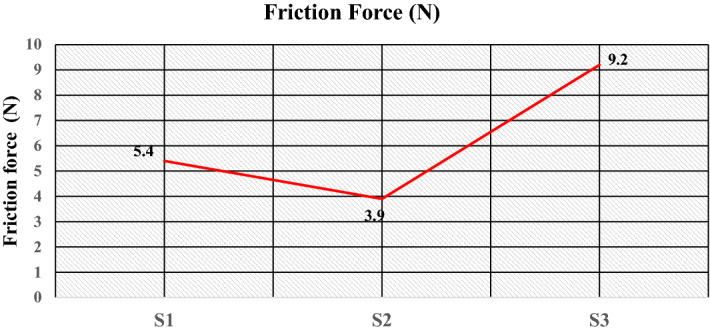
Friction force of the specimens.

**Figure 20:**
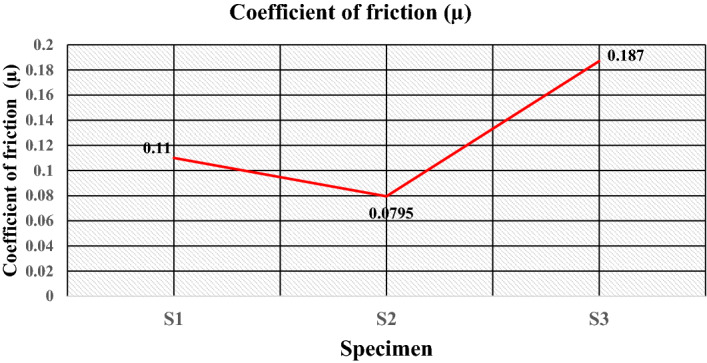
Coefficient of friction of the specimens.

**Figure 21:**
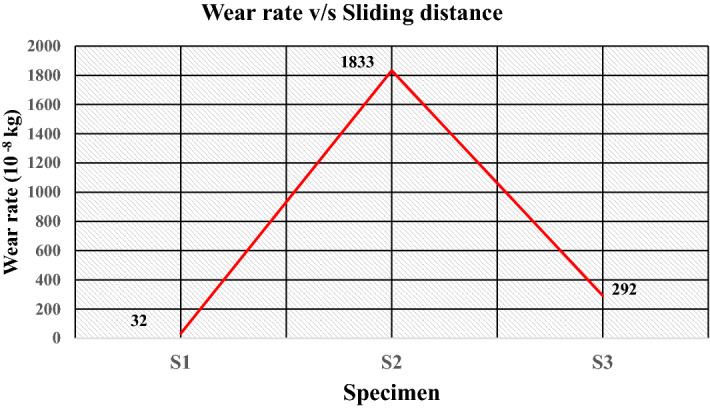
Wear rate of the specimens at sliding distance of 226 m.

Figure 18a–c gives the plot of the friction force at a load of 5 kgf, i.e., 49.05 N during sliding wear, which shows that the value of the friction force during sliding wear fluctuates because of the change in the surface properties during the sliding wear. The optimum value of the friction force plotted in Fig. 19 shows that specimen S1 attains a friction force of 5.4 N. This value decreases to 3.9 N for specimen S2 and increases to 9.2 N for specimen S3. The coefficient friction is calculated by dividing the friction force (F) by the normal reaction (R), which is plotted in Fig. 20. It indicates that the coefficient of friction varies from 0.0795 to 0.11. The deboning of the matrix and fillers and the transfer of the matrix affects the surface characteristics at the pin disc interface and influence the coefficient of friction. The wear is calculated for the given sliding distance of 226 m, as shown in Fig. 21. This indicates proportionality to the wear rate with minimum and maximum wear of 32 × 10^–8^ and 1833 × 10^–8^ (kg), respectively.

**Figure 22:**
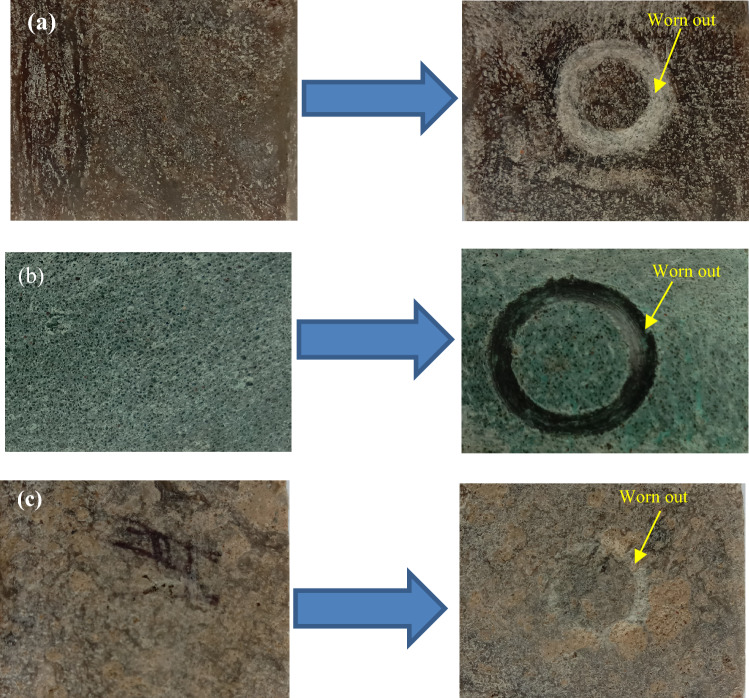
Image of the worn-out surfaces (**a**) S1, (**b**) S2, (**c**) S3.

The wear rate (kg/m) indicates loss in weight (kg) for unit sliding distance (meter). The images of the test specimens are given in Fig. 22a–c, which shows the grooves are formed due to the pin’s sliding actions over the specimen's surfaces. The wear rate for the prepared specimens for floor tiles at the given loads is given in Table 6. The wear behaviour of the material during sliding is complex due to the interdependency of several encountering forces and varying properties. The wear rate is dependent on hardness, ductility, elastic stiffness, plasticity, strain, strength, toughness, and viscosity^56,57^. Micro-cutting, micro-ploughing, fibre peelings and deboning were identified as the possible wear mechanisms for the tested polymeric composites. Moreover, on increasing load, wedge formation was observed for specimen S3.

**Figure 23:**
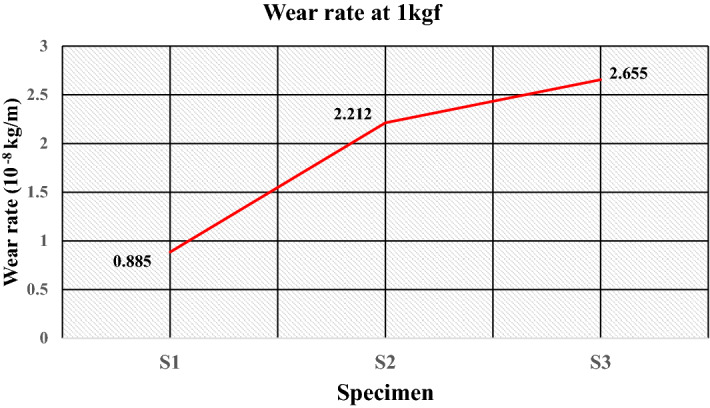
Wear rate of the specimens at 1 kgf.

**Figure 24:**
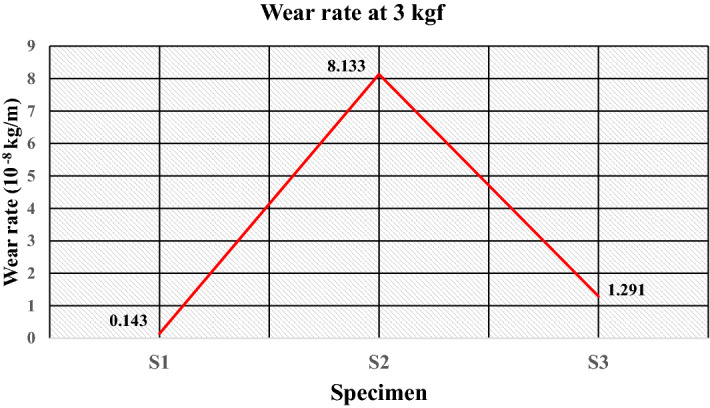
Wear rate of the specimens at 3 kgf.

The plot of the wear rate (kg/m) at 1 kgf is given in Fig. 23, which shows that specimen S1 obtains a minimum wear rate of 0.885 × 10^–8^ kg/m. The good elasticity of LDPE and a sufficient number of filler particles also result in the load being insufficient to cause adhesion of the matrix resulting in a minimum wear rate. The wear rate increases to 2.212 × 10^–8^ kg/m for specimen S2 because of reduced elasticity by replacing the matrix with HDPE. Moreover, the lower fraction of filler particles and insufficient causes maximum wear of 2.655 × 10^–8^ kg/m for specimen S3. The values of the wear rates under the load of 3 kgf are illustrated in Fig. 24. It shows that specimen S1 having compositions of 50% of low-density polyethylene and 50% silica sand particles, has a minimum wear rate of 0.143 × 10^–8^ kg/m, which reveals the incorporation of silica sand particles improves wear resistance as fillers. Hard sand particles are believed to improve the sliding wear resistance between the rubbing pairs. The wear rate is further increased to 1.291 × 10^–8^ kg/m for specimen S3 with the reduced fraction of fillers having 30 wt% of sand particles and 70 wt% as waste plastics (50 wt% of LDPE and 20 wt% of PET) with a wear rate of 1.291 × 10^–8^ kg/m.

**Figure 25:**
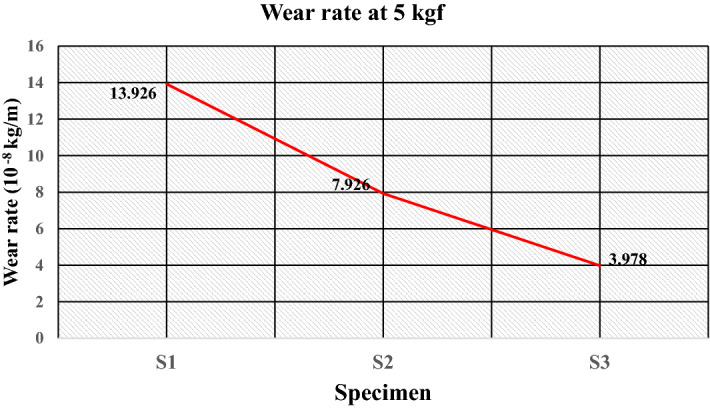
Wear rate of the specimens at 5 kgf.

Furthermore, despite the better mechanical properties, the maximum wear rate is found for specimen S2 to be 8.133 × 10^–8^ kg/m, having compositions of 50 wt% of the high-density polyethylene and 50% of sand particle. The high friction coefficient at the surface of the specimen could be the possible cause of the behavior. The plot of the wear rate (kg/meter) at 5 kgf is given in Fig. 25, which shows that specimen S1 obtains a maximum wear rate of 13.926 × 10^–8^ kg/m. The lower strength of LDPE compared to HDPE and PET and the load sufficient for deboning the filler results in a maximum wear rate. The wear rate decreases to 7.926 × 10^–8^ kg/m for specimen S2 because of the increased strength of the matrix as HDPE. The wear rate (kg/m) is further reduced to 3.978 × 10^–8^ kg/m for specimen S3 because of the strength and elasticity provided by the matrices such as LDPE and PET.

## Conclusion

The study highlights the challenges and issues associated with solid waste management and examines the opportunities for effective utilization of generated solid waste. The experimental work successfully demonstrates the manufacturing of floor tiles by using different types of waste plastics and silica sand in proportions. From the literature, various characterization techniques are important for successfully recycling plastic waste for the development of sustainable building materials such as floor tiles. The method of utilizing waste plastics in construction industries reduces the requirement for fresh raw material, lowering construction costs while also improving the environmental condition. The conclusions drawn from experimental investigations are as follows:

1. The microstructural analysis observes sufficient interfacial adhesion, and the distribution of silica sand is uniform, confirming the mixture’s homogeneity.
2. The percentage of water absorption for the developed floor tile specimens is quite low i.e. < 0.5% (as per ASTM C373 for floor tiles) thus, making it suitable for use under different ambient conditions, i.e., dry, wet or moisture.
3. The maximum compressive strength and flexural strength were obtained for the specimen having 50 wt% of high-density polyethylene and 50 wt% of sand as composition at 46.20 N/mm^2^ and 6.24 N/mm^0^, respectively. The increased interfacial bonding due to increased surface area could be the possible cause of the improvement in mechanical strength.
4. The ranges of wear rate (kg/m) for the developed samples at load of 1 kgf, 3 kgf and 5 kgf were found to be 0.885–2.655, 0.143–1.291 and 3.978–13.926 (10^–8^ kg/m) respectively, which is less than 0.0001 g/m therefore satisfies the ASTM C501 standard for wear of floor tiles. Moreover, for a floor tile it is recommended to have a minimum coefficient of friction of 0.7 as per ASTM C 1028. In the present study, the friction force and coefficient of friction were found in the ranges of 3.9–9.2 and 0.0795–0.187 respectively. Therefore, tribological behaviour is satisfactory for its use as floor tiles. However, invariability is existing due to the interdependency of several encountering forces and varying properties.
5. The results of the different properties for the developed tile specimens were found sufficient for use as paving material in non-traffic areas of public places.

## Data Availability

All data generated or analyzed during this study are included in this published article.
